# Strategy to Enhance Influenza Surveillance Worldwide^1^

**DOI:** 10.3201/eid1508.081422

**Published:** 2009-08

**Authors:** Justin R. Ortiz, Viviana Sotomayor, Osvaldo C. Uez, Otavio Oliva, Deborah Bettels, Margaret McCarron, Joseph S. Bresee, Anthony W. Mounts

**Affiliations:** University of Washington, Seattle, Washington, USA (J.R. Ortiz); Ministerio de Salud, Santiago, Chile (V. Sotomayor); Instituto Nacional de Epidemiología, Mar del Plata, Argentina (O.C. Uez); Pan American Health Organization, Washington, DC, USA (O. Oliva); Centers for Disease Control and Prevention, Atlanta, Georgia, USA (D. Bettels, M. McCarron, J.S. Bresee, A.W. Mounts)

**Keywords:** influenza, human influenza influenza A virus, avian influenza, H5N1 subtype, sentinel surveillance, epidemiology, viruses, policy review

## Abstract

The emergence of a novel strain of influenza virus A (H1N1) in April 2009 focused attention on influenza surveillance capabilities worldwide. In consultations before the 2009 outbreak of influenza subtype H1N1, the World Health Organization had concluded that the world was unprepared to respond to an influenza pandemic, due in part to inadequate global surveillance and response capacity. We describe a sentinel surveillance system that could enhance the quality of influenza epidemiologic and laboratory data and strengthen a country’s capacity for seasonal, novel, and pandemic influenza detection and prevention. Such a system would 1) provide data for a better understanding of the epidemiology and extent of seasonal influenza, 2) provide a platform for the study of other acute febrile respiratory illnesses, 3) provide virus isolates for the development of vaccines, 4) inform local pandemic planning and vaccine policy, 5) monitor influenza epidemics and pandemics, and 6) provide infrastructure for an early warning system for outbreaks of new virus subtypes.

## Abstract 2

Sentinel surveillance for severe acute respiratory infection and influenza-like illness is effective in resource-limited settings.

The emergence of a novel strain of influenza virus A (H1N1) in April 2009 and its subsequent rapid global spread have focused attention on influenza surveillance capabilities worldwide (*1*). A consultation convened by the World Health Organization (WHO) in 2005 had previously concluded that the world was unprepared to respond to an influenza pandemic, due in part to inadequate global surveillance and response capacity (*2*). The International Health Regulations 2005 call for strengthened surveillance for all events that may constitute a “public health emergency of international concern”; such events include individual human cases of influenza caused by a new subtype of influenza virus A (*3*). As part of the International Health Regulations 2005 core surveillance and response capacity requirements, each Member State must develop and maintain capabilities to detect, assess, and report disease events nationally and internationally to WHO within 48 hours of confirmation. However, reviews of national pandemic planning indicate that surveillance systems are often inadequate to support current preparedness strategies (*4*–*8*). WHO has existing surveillance guidelines to help Member States implement universal surveillance for novel and pandemic influenza (*9*), but the guidelines lack the specificity that would enable many countries to establish operational surveillance plans.

Quality influenza surveillance systems are needed to enable countries to better understand influenza epidemiology, including disease incidence and severity, and help them implement appropriate prevention strategies. The challenges experienced by the United States and Mexico to rapidly determine the extent and severity of illness of the 2009 novel influenza A (H1N1) outbreak highlighted the need for systems that can reliably produce these estimates. Furthermore, global strategies to address other vaccine-preventable diseases have acknowledged the importance of establishing local disease burden (effects, severity, amount of illness, and costs) as a first step toward decisions about the introduction of vaccines into new countries. We describe a generic guideline for collecting data on severe acute respiratory infection (SARI), influenza-like illness (ILI), and laboratory-confirmed influenza that can be implemented in limited-resource settings.

## Current Situation

### Global Influenza Surveillance

For 60 years, the WHO Global Influenza Surveillance Network (GISN) has provided virologic information used in the biannual process of selecting strains for the Northern and Southern Hemisphere influenza vaccine formulations. However, its capacity to provide epidemiologic data or an alert of an emerging pandemic is limited. GISN currently comprises 122 National Influenza Centers in 87 countries and 4 WHO Collaborating Centers for Reference and Research on Influenza (*10*). Although this system has proven to be valuable, tropical and resource-limited countries (particularly in Africa) are underrepresented (*11*).

### Influenza in Developing Countries

Virus transmission or clinical presentation may be altered by differences in cultural practices, the environment, geography, human genetics, and social structures. Enhanced influenza surveillance can permit assessment of a number of factors that may affect disease activity: population density, differences in prevalence and spectrum of chronic illness, proximity of the young and elderly, low proportion of elderly in the population, low school attendance, and school schedules that may not correspond with peak transmissibility season. The effectiveness of control measures such as social distancing and vaccination may differ between developed and developing settings because of these factors.

Available epidemiologic evidence suggests that influenza is common in tropical regions and contributes substantially to disability and use of healthcare resources (*12*–*16*). Data describing the seasonality and epidemiology of influenza in tropical areas are limited; however, some tropical countries report year-round human influenza activity (*12*), unlike in temperate regions where transmission occurs with marked seasonality. Because of these limited data, most of the understanding of seasonal influenza is derived from epidemiologic data collected in western Europe and North America. Nevertheless, estimates of a pandemic impact indicate that most deaths will be in developing countries and that more than half will occur in southern Asia and sub-Saharan Africa (*17*). A better understanding of the epidemiology of influenza in these areas would facilitate country-appropriate pandemic planning and vaccine policy development.

## Objectives

The most efficient process for producing high-quality epidemiologic data for influenza-associated illness is sentinel surveillance. The primary limitation of most existing influenza sentinel-site networks that track ILIs has been that they often provide little epidemiologic data, do not produce data on disease incidence, and are focused on mild disease, which supports the notion that influenza is a benign disease. We propose that influenza surveillance should capture severe influenza outcomes as a primary measure. Hospital-based sentinel surveillance is the most efficient way to collect clinical data and laboratory specimens from persons with a prevalent and severe infectious disease.

Carefully placed sentinel sites can provide adequate information on the epidemiology of influenza without the need for comprehensive national case ascertainment or reporting. Placing surveillance sites where population data are known would permit calculation of population-based estimates of disease rates according to age and other demographic variables. In addition, collection of clinical specimens from persons from whom epidemiologic data are also collected would ensure virus strain surveillance and provide isolates that can be used for vaccine development.

A sentinel surveillance system can be used to monitor >1 disease, can be sustainable, and can integrate with and build upon existing systems. The system objectives are 1) describe the disease impact and epidemiology of severe, acute, febrile respiratory illness and define the proportion that is associated with influenza; 2) provide influenza virus isolates for monitoring changes in viral antigens and development of new vaccines; 3) contribute data for local pandemic planning and making decisions regarding vaccine policy; 4) provide infrastructure for an early warning system for outbreaks of new subtypes of influenza A viruses and new strains of existing subtypes; and 5) serve as a monitoring tool for pandemic influenza.

## Components and Processes

### Case Definitions

These surveillance guidelines use the existing WHO case definition for ILI and incorporate WHO guidance to define SARI in adults and children (Table 1). The case definitions fit within the existing framework for pandemic early warning, use existing definitions for ease of adoption, and rely on physical examination findings that do not require laboratory or radiographic criteria. In addition, SARI definitions may capture a broad spectrum of severe influenza-associated illness, including exacerbations of asthma, chronic obstructive pulmonary disease, and decompensated congestive heart failure, which may account for ≈75% of hospitalized influenza patients (*16*,*20*,*21*).

**Table 1:** Influenza sentinel surveillance case definitions*

Case	Definition criteria
Influenza-like illness	ALL OF THE FOLLOWING • Sudden onset of fever >38°C, AND • Cough or sore throat, AND • Absence of other diagnoses
Severe acute respiratory infection in persons >5 years of age	ALL OF THE FOLLOWING • Sudden onset of fever >38°C, AND • Cough or sore throat, AND • Shortness of breath or difficulty breathing, AND • Requires hospitalization
Severe acute respiratory infection in persons <5 years of age	EITHER IMCI criteria for pneumonia Any child 2 mo to 5 y of age with cough or difficult breathing and: • breathing faster than 60 breaths/min (infants <2 mo) • breathing faster than 50 breaths/min (2–12 mo) • breathing faster than 40 breaths/min (1–5 y) OR IMCI criteria for severe pneumonia Any child 2 mo to 5 y of age with cough or difficult breathing and any of the following general danger signs: • unable to drink or breastfeed • vomits everything • convulsions • lethargic or unconscious • chest indrawing or stridor in a calm child AND Requires hospital admission

*Surveillance guidelines use the existing World Health Organization (WHO) case definition for Influenza-like Illness (*19*), and incorporate WHO guidance to define severe acute respiratory infection in adults and children (*9*,*18*,*19*). IMCI, Integrated Management of Childhood Illness.

### Sentinel Site Selection

Ideally, sites should represent a wide cross-section of ethnic and socioeconomic groups and should be in different climatic regions. Placement of sites in areas where the population denominator can be ascertained or estimated will facilitate incidence estimates. Ultimately, the choice of sentinel hospitals will often be based on practical issues such as human resources, communication infrastructure, and availability of specimen transport and testing. There is no ideal number of surveillance sites; the number chosen by a particular country will depend in part on sustainability and resources available.

### Data Collection

Minimum data elements are outlined in Table 2. Data collected should be adequate for routine public health surveillance and description of key epidemiologic features of disease. Data can be broadened to include clinical signs and symptoms, potential exposures, laboratory data, and therapies.

**Table 2:** Sample data collection from cases of severe acute respiratory infection and influenza-like illness*

Recommended essential minimum data for SARI surveillance
General information
• Unique identification number
• Medical record number
• Name (of patient and parent’s name, if a minor)
• Date of birth
• Sex
• Address
• Date of onset of symptoms
• Date of collection of epidemiologic data
• Suspected novel influenza case
• Inpatient or outpatient
Clinical signs and symptoms
• Fever >38°C
• Cough
• Sore throat
• Shortness of breath/difficulty breathing
• Other clinical danger signs (*19* *22**,**23**,*)
Type of specimen collected and date of collection
• Throat swab specimen, date of collection
• Nasal swab specimen, date of collection
• Other specimen (if collected), date of collection
Preexisting medical conditions
• Liver disease
• Kidney disease
• AIDS, cancer, or other immunocompromised state
• Neuromuscular dysfunction
• Diabetes
• Heart disease
• Lung disease
• Smoking history
Optional data collection for SARI surveillance
General information
• Diarrhea
• Encephalopathy
Exposure
• Occupation of patient
• Part of an outbreak investigation
• Contact with sick or dead poultry or wild birds
• Contact with friend or family who has SARI
• Travel in an area known to have endemic circulation of avian influenza (H5N1)
• Other high-risk exposure (e.g., eating raw or undercooked poultry products in an area of influenza virus [H5N1] circulation)
Vaccine/treatment history
• Vaccination against influenza within the past year
• Currently taking antiviral medicine

*SARI, severe acute respiratory infection; ILI, influenza-like illness.

### Specimen Collection

Respiratory specimens should be collected early from all SARI patients, following established protocols (*24*). If resources do not allow collection from all patients, an unbiased systematic sampling scheme should be established. To develop quality estimates of incidence and severity, data and specimens from all or most SARI patients from a few facilities would be preferred over a small sample of SARI patients from multiple facilities.

Because seasonality, attack rates, and public health priorities differ from country to country, there is no generic number of specimens to be collected by each site. The number must be determined by the primary surveillance objective (e.g., understanding of seasonality, risk factor analysis, or determination of clinical outcomes) and must represent climatic and geographic regions. For example, a country with coastal, mountainous, and tropical regions may have different influenza activity in each region and may thus require more surveillance sites and increased specimen collection than neighbors or similarly sized countries. Therefore, the number of specimens collected must be approached on a case-by-case basis and depends on objectives of a country, country-specific geographic and climatic issues, and public health priorities.

### Integration into National Reporting Systems

In countries with established national disease reporting systems, such as the Integrated Disease Surveillance Reporting system used in Africa (*25*), sentinel surveillance for SARI can be incorporated into the existing system. Because Integrated Disease Surveillance Reporting is generally a passive surveillance program, a few select sites should serve as embedded sentinel sites; intensive training and close follow-up should be conducted to ensure the quality of the reported data.

### Outpatient Surveillance

The highest priority should be to collect data on SARI cases because they contain the most influenza-associated disability and premature death. However, if resources permit, data collection at sentinel sites should be expanded to include ambulatory patients with ILI. Because the number of cases at ambulatory care sites is likely to be large, case counts would be aggregated, and clinical specimens and epidemiologic data would be collected from only a small sample of patients. Weekly case counts should be categorized by age group according to well-studied age-range categories (6–23 months, 2–4 years, 5–17 years, 18–49 years, 50–64 years, and >65 years) (*26*). Patients chosen to give detailed epidemiologic data and clinical specimens should be selected in as unbiased a manner as possible. The selection protocol must take into account local health-seeking behavior, such as differential use of evening and weekend clinics. Ideally, the weekly total number of patients seen by clinics would also be collected by age group to allow for proportion of ILI to be calculated. Rapid system expansion can compromise the quality of collected data; therefore, ILI surveillance should emphasize quality data collection from a few well-run sites.

### Laboratory Testing

Clinical specimens should be collected from a high proportion of SARI patients and a systematic sample of ILI patients. These specimens can be processed in sentinel site laboratories, but further analyses may require their transport to additional laboratories. Ideally, specimens would be tested for evidence of influenza viruses by reverse transcription–PCR (RT-PCR). A subset of specimens should undergo viral culture and antigenic characterization. Surveillance data should be submitted to WHO FluNet, and, if possible, national laboratories should work with a WHO Collaborating Center laboratory to submit sample virus isolates for vaccine strain selection.

In countries where influenza spreads in seasonal epidemics, it may be adequate to collect less epidemiologic data and fewer specimens for laboratory testing by sampling a smaller proportion of SARI patients during the noninfluenza season. Knowledge of SARI rates outside influenza season will permit comparisons between peak season and baseline rates. Non–influenza season rates of SARI can also be monitored by public health authorities, because anomalies in SARI rates could represent outbreaks in need of investigation. However, high-quality, year-round data will be required for >1 season before assumptions can be made about seasonality in a region.

Nasal and nasopharyngeal specimens have a higher yield for influenza virus detection in ILI cases than do oropharyngeal specimens (*27*). However, the relative sensitivity of nasal versus oropharyngeal swabs to detect influenza virus infection in SARI cases is unknown. If both are collected, specimens can be placed in the same tube of viral transport media for processing. If SARI patients are intubated, endotracheal aspirates can also be used. Specimens can be frozen at –70°C for storage and possible future assessment of other respiratory pathogens.

The sensitivity and specificity of any test for influenza will depend on the laboratory performing the test, the quality of the clinical specimen, the manner in which the specimen is processed, and the type of specimen collected. Generally, RT-PCR testing of respiratory specimens is the most sensitive laboratory test for influenza virus, but it is relatively expensive and is not useful for antigenic characterization (*28*). If the proper primers and probes are used, RT-PCR can determine influenza virus A subtype and can detect novel influenza virus A subtypes. Fluorescent antibody tests, although less expensive, are less sensitive and specific than RT-PCR (*27*). Rapid point-of-care tests are less sensitive and specific than RT-PCR or fluorescent antibody tests and are not generally recommended for use by sentinel surveillance. Virus culture has been the diagnostic standard for identifying influenza virus. Culture sensitivity depends on proper specimen handling and the experience of the laboratory. Virus culture should be performed on at least a sample of specimens to provide material for antigenic determination and potential isolates for vaccine production.

## Data Analysis and Reporting

Timely analysis and reporting of surveillance data will facilitate treatment decisions by clinicians and control measures by public health officials. It will also encourage continued reporting of cases by clinicians in the surveillance system. Weekly reports of clinical and laboratory confirmed case counts should be disseminated throughout the surveillance system to participating healthcare providers and all stakeholders during peak seasons. The frequency of reports and the extent to which they are disseminated will depend on data timeliness and public health priorities. Sentinel surveillance reporting mechanisms should use existing public health communications systems and augment other reporting mechanisms such as FluNet through WHO GISN (*29*).

Basic analyses of surveillance data should include weekly frequencies of SARI and laboratory-confirmed influenza cases as well as the proportion of tested patients, by age group, who are influenza virus positive. If possible, proportions of SARI and influenza cases per total of weekly sentinel hospital admissions should be reported. Reports with case frequencies and proportions during prior weeks and years will demonstrate trends over time. At least once annually, analyses of surveillance data to determine risk factors for disease should be reported. These reports should use collected data on concurrent conditions and population-based rates, if these can be determined.

Understanding the epidemiology of severe influenza-associated disease is essential for decisions related to vaccine recommendations. These data are prioritized in the guidelines because many developing countries have limited funds and competing healthcare priorities. However, data collected during SARI surveillance alone will be inadequate to describe aspects of influenza epidemiology such as transmission dynamics, costs, and occurrence of mild disease.

## Evaluation and Quality Assurance

The usefulness of surveillance data will depend directly on the quality of the data; every system should have a quality assurance program. Quality indicators will reflect such attributes as system acceptability, timeliness, completeness, and representativeness of collected data. These attributes should be assessed routinely. In addition, the system should undergo regular data audits and systematic field evaluation. In 2001, the Centers for Disease Control and Prevention published comprehensive guidelines for the evaluation of public health surveillance systems (*30*). These guidelines serve as a template for sentinel surveillance evaluation and quality recommendations. Several key quality indicators are recommended in the following section and in Table 3.

**Table 3:** Influenza surveillance evaluation and recommended quality indicators*

1. Timeliness
a. Several time intervals are appropriate for routine measurement as quality indicators. These include the duration of time from
i. Target date for data reporting from the sentinel site to the next administrative level until the actual reporting date
ii. Target date for data reporting from the next administrative level to the national level until the actual reporting date
iii. Date of specimen collection at facility until shipment to laboratory
iv. Date of result availability in laboratory until date of report to referring institution and physician
v. Date of receipt of specimen in the laboratory until result availability
b. Metrics. Two metrics can be used to reflect timeliness indicators:
i. Percentage of time that a site achieves target for timeliness
ii. Average number of days for each interval over time for each site
2. Completeness
a. Percentage of reports received from each site with complete data
b. Percentage of data reports that are received
c. Percentage of reported cases that have specimens collected
3. Audit. Regular field evaluations and audits at facility level of a subset of medical records to ensure
a. Cases are being counted appropriately and not being underreported
b. Reported cases fit the case definition
c. Epidemiologic data are correctly and accurately abstracted
d. Respiratory samples are being taken, stored, processed, tested, and shipped properly and in a timely fashion from all those who meet sampling criteria
e. Sampling procedures are being done uniformly without evidence of bias
4. Data to be followed and observed for aberrations over time
a. Number of cases reported by month for each site
b. Number of specimens submitted by month for each site
c. Percentage of specimens that are positive for influenza
d. Number and percent of ILI and SARI cases tested

*ILI, influenza-like illness; SARI, severe acute respiratory illness.

### Data Validity

Regular field evaluations and audits at a facility level must be a standard component of the system. This process can determine that cases are being counted appropriately, that reported cases meet the case definition, and that sampling procedures are being used uniformly without evidence of bias. Data values recorded in the surveillance system can be compared with standard chart-review values by a retrospective review of a sample of medical records. If a sampling procedure is used for specimen collection, audits can ensure that procedures are uniform and unbiased. Additionally, audits can determine whether clinical specimens are being taken, stored, processed, tested (if appropriate), and shipped properly and in a timely manner from all those who meet sampling criteria.

Observance of expected trends in reporting and disease activity can provide an additional means of assessing data quality. Although it is not possible to define expected values for some parameters, such as the percentage of specimens testing positive for influenza virus or the number of SARI cases occurring in a given facility, aberrations in the data over time or substantial differences between facilities can signal problems at a given site. Trends assessed may include number of cases reported by month, number of specimens submitted by month, percentage of influenza-positive specimens, and number and percentage of SARI and ILI cases tested.

### Timeliness

To be useful, collection and reporting of surveillance data must be timely. Timeliness of the following activities is appropriate for routine measurement as quality indicators for surveillance sites: data reporting, specimen shipment to the laboratory for testing, receipt of specimens by the laboratory, laboratory processing and testing of specimens, and reporting of laboratory results.

One way to quantify timeliness is to calculate the percentage of times that a site achieves targets for specific intervals, for example, the percentage of times that a site sends reports or specimens to the appropriate place within a specified time frame. A hypothetical system may choose as a goal that 80% of data reports be sent within 48 hours of the reporting deadline or that 80% of specimens be shipped within 48 hours of specimen collection. Likewise, for the laboratory, the percentage of samples that are tested and have final results within a target time frame can be calculated. Targets will depend on site-specific circumstances and public health priorities.

A similar quality metric that can be used is the calculation of the average time to accomplish surveillance activities. For example, a hypothetical site that is chronically late in sending data every month might average several days between the deadline for receipt (the day of the week or month on which reports are due) and actual receipt of data. For laboratory specimen processing, the average number of days between receipt of specimens and the reporting of the results can be measured and followed similarly. Site time averages can be compared to identify sites that are underperforming and to target improvements. Either percentages of sites achieving timeliness targets or time lag averages can also be used as a quality metric to be followed over time.

### Completeness

Indicators of completeness can be determined by analyzing reported data. They may include percentage of reports received from each site with complete data, percentage of total expected data reports received, and percentage of total expected cases that have specimens submitted to the laboratory (depends on sampling scheme devised for sites).

## Pandemic Early Warning Systems and Monitoring

Emergence of new subtypes of influenza virus A in human populations is unusual and unlikely to be detected by a sentinel surveillance system, except by chance or if transmission is sustained. Control of a pandemic caused by the introduction of a new subtype of influenza virus A will require early detection and recognition of the event. Although sentinel surveillance as a stand-alone system may not accomplish this, it has value in establishing the infrastructure necessary to respond to a pandemic. In addition to providing a basic understanding of the epidemiology of influenza transmission and risk, a routine reporting system would produce an infrastructure for reporting, specimen processing and testing, and data collection and analysis. It would make data interpretation more routine (and thus more manageable in the face of a pandemic emergency) and drive interest in influenza-associated disease and vaccination.

After a novel strain of influenza emerges, monitoring its course is necessary to determine whether cases are increasing or decreasing, to detect changes in patient age distribution or other epidemiologic characteristics, to detect changes in mortality rates, and to monitor changes in susceptibility to antiviral agents. In the midst of an outbreak, national monitoring may not be necessary or feasible, and most, if not all, critical information can be gained from a few sentinel sites. Emergence of a new strain of influenza increases the data needs of health policy makers. Historical surveillance data for comparison can facilitate the understanding of answers to critical questions such as severity of the outbreak related to a new strain and its potential to adversely affect healthcare delivery. An existing surveillance infrastructure also provides the platform needed to describe the clinical course of emerging pathogens, risk factors for severe outcomes, and effectiveness of control measures.

## Conclusions

Surveillance for SARIs can provide critical understanding of the contribution of influenza infection to the global burden of disease, provide a platform for the study of other common respiratory pathogens, and strengthen public health infrastructure. Such a system should be a part of a routine surveillance program to provide data needed for allocation of scarce healthcare resources.
